# The *p53* mRNA: an integral part of the cellular stress response

**DOI:** 10.1093/nar/gkz124

**Published:** 2019-03-04

**Authors:** Lucia Haronikova, Vanesa Olivares-Illana, Lixiao Wang, Konstantinos Karakostis, Sa Chen, Robin Fåhraeus

**Affiliations:** 1 RECAMO, Masaryk Memorial Cancer Institute, Zluty kopec 7, 656 53 Brno, Czech Republic; 2 Laboratorio de Interacciones Biomoleculares y cáncer. Instituto de Física Universidad Autónoma de San Luis Potosí, Manuel Nava 6, Zona universitaria, 78290 SLP, México; 3 Department of Medical Biosciences, Umeå University, 90185 Umeå, Sweden; 4 Inserm U1162, 27 rue Juliette Dodu, 75010 Paris, France; 5 ICCVS, University of Gdańsk, Science, ul. Wita Stwosza 63, 80-308 Gdańsk, Poland

## Abstract

A large number of signalling pathways converge on p53 to induce different cellular stress responses that aim to promote cell cycle arrest and repair or, if the damage is too severe, to induce irreversible senescence or apoptosis. The differentiation of p53 activity towards specific cellular outcomes is tightly regulated via a hierarchical order of post-translational modifications and regulated protein-protein interactions. The mechanisms governing these processes provide a model for how cells optimize the genetic information for maximal diversity. The *p53* mRNA also plays a role in this process and this review aims to illustrate how protein and RNA interactions throughout the *p53* mRNA in response to different signalling pathways control RNA stability, translation efficiency or alternative initiation of translation. We also describe how a *p53* mRNA platform shows riboswitch-like features and controls the rate of p53 synthesis, protein stability and modifications of the nascent p53 protein. A single cancer-derived synonymous mutation disrupts the folding of this platform and prevents p53 activation following DNA damage. The role of the *p53* mRNA as a target for signalling pathways illustrates how mRNA sequences have co-evolved with the function of the encoded protein and sheds new light on the information hidden within mRNAs.

## INTRODUCTION

**Figure 1.**
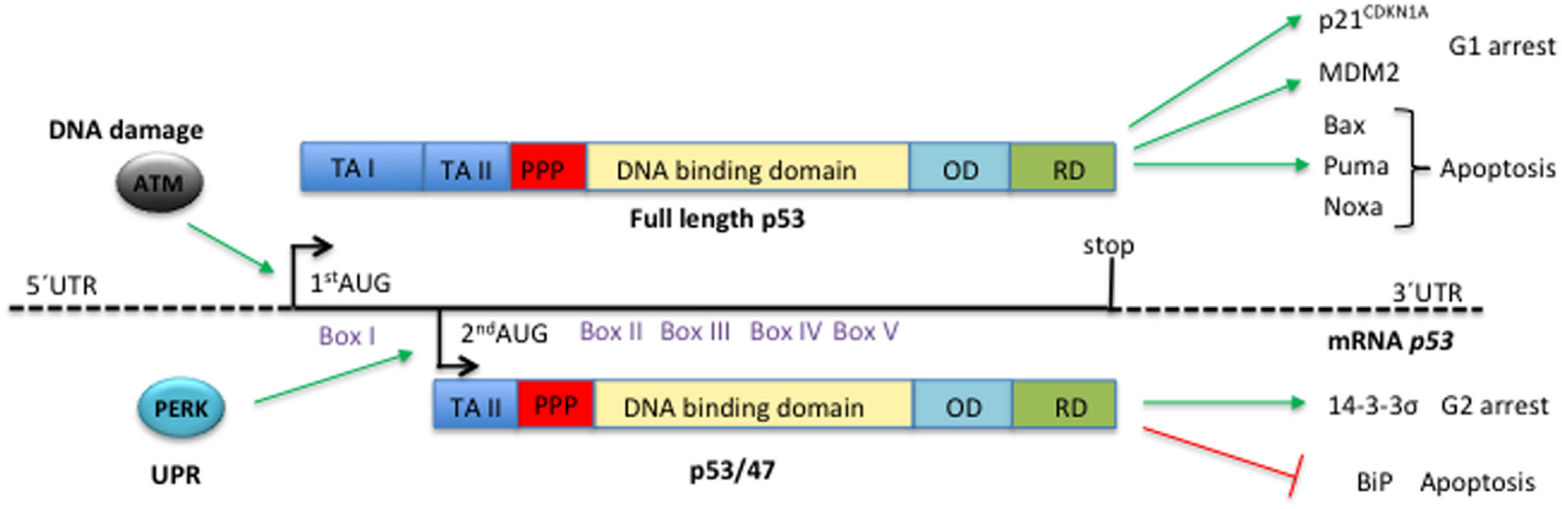
*Cell stress-dependent regulation of alternative initiation of p53 mRNA translation*. Alternative translation initiation of the *p53* mRNA generates two isoforms, p53 full length (p53FL) and p53/p47. The activation of the ATM kinase (Ataxia Telangiectasia Mutated) following DNA damage results in the induction of p53FL synthesis from +1 AUG. The full-length p53 includes the TA I (transactivation domain I) that is required for induction of p53 target genes, including the G1 cell cycle kinase inhibitor p21^CDKN1A^ or pro-apoptotic factors such as Bax, Puma or Noxa of the Bcl-2 family, to mention just a few. The activation of the Unfolded Protein Response (UPR) pathway following stress to the endoplasmic reticulum activates the PERK kinase and the initiation of the p53/47 isoform at the second in frame AUG at +120. P53/47 lacks TA I but retains TA II and causes G2/M arrest via induction of 14-3-3σ, or a BIK-dependent apoptosis by suppressing the BiP chaperone. Apart from the *box I*, the *p53* mRNA harbours four other conserved domains (*II* to *V*) within the DNA binding domain (164). Different p53 protein functional domains are indicated: trans-activation domains I & II (TA I & II); poly-proline rich region (PPP); oligomerization domain (OD); regulatory domain (RD). For a more comprehensive overview of signalling pathways activating p53 please see (3,4).

The p53 tumour suppressor protein is activated in response to various cellular stresses such as the DNA damage and the unfolded protein response (UPR) pathways (for review see (1–4)). The appropriate cell biological response to the causing damage depends on cell type, intensity and duration of the stress and is the result of altered expression of some of p53’s several hundred target genes. This includes genes associated with cell cycle progression through G1 or G2, metabolic pathways and cellular repair, or irreversible factors that induce apoptosis or cellular senescence (Figure 1). p53 is regarded as a tumour suppressor but it also harbours pro-survival and growth-promoting activities revealed by ‘gain of function’ mutations of p53 (4). One of the outstanding questions regarding p53 activation is how different cell types control the multifunctional aspects of p53 in response to changes in cellular conditions. The differentiation of p53 activity includes post-translational modifications that regulate intrinsically disordered domains which provide interfaces for a large number of proteins (5,6). In this way, p53 can select binding partners according to the signalling pathway. The expression of isoforms with specific activities that can form homo- or hetero-oligomers provides an additional level of differentiation (7). The *p53* mRNA also plays a role in regulating p53 activity and this review focuses on different ways by which the *p53* mRNA helps differentiate p53-mediated response to signalling pathways. We describe how the *p53* mRNA affects post-translational modifications and the stability of the nascent protein as well as the expression of p53 isoforms with unique functions.

One of the first reports indicating a stress-dependent regulation of *p53* mRNA translation came from Kastan *et al.* showing an mRNA translation-dependent increase of newly synthesized p53 proteins following DNA damage without a corresponding increase in mRNA levels (8). It was later observed that more *p53* mRNA was associated with polysomes following γ-irradiation (9). Starting from there, we will present what is known about the *p53* mRNA today and highlight its crucial role in p53 stress response pathways.

The rate of mRNA translation is encrypted within the transcript and determined by its interactions with cellular factors. Besides the main basic elements such as the 5′m^7^G cap and the poly(A) tail that provide general mechanisms of translation initiation, other regulatory elements of the untranslated (UTRs) and the coding regions provide specificity and fine-tuning of protein synthesis (10,11). However, protein expression levels not only depend on synthesis but equally on protein degradation and the *p53* mRNA also harbours information that helps control p53 protein turnover rate.

Synonymous mutations are known to affect the encoded protein but the role of codon changes in cell biological processes are often overlooked. With the exception of cases when pre-mRNA splicing is involved, the cell biological effects of alternative codons is usually attributed to changes in protein folding due to changes in the rate of translation elongation followed by altering ‘fast’ and ‘slow’ codons (12–14). Here we describe an alternative mechanism whereby a single nucleotide change in the *p53* mRNA coding sequence affects the folding of the RNA and how this has consequences for the stability and the activity of the encoded protein. Finally, we describe how a *p53* mRNA structure has evolved from temperature-dependent regulation in pre-vertebrates to a chaperone-mediated stress-response riboswitch in mammalian cells.

### The role of the *p53* mRNA 3′ UTR

The 3′ UTRs play an important role in controlling mRNA stability, localization, translation and degradation (15). Generally, the processing of the 3′ end of primary transcripts takes place in the nucleus and up to 85 proteins are suggested to be involved in the three main steps: endonucleolytic cleavage, polyadenylation and export (16). In addition to nuclear polyadenylation, a cytoplasmic polyadenylation may occur due to the presence of specific sequences in 3′ UTR called ‘cytoplasmic polyadenylation elements’ (CPEs) (17). Many eukaryotic mRNAs contain *cis*-acting regulatory elements in their 3′ UTRs that can act as targets for trans-acting elements (proteins or RNA). The most abundant *cis*-elements in eukaryotic mRNAs is the AU-rich element (ARE), which *per se* promotes RNA instability (18) but can also have indirect positive effects on RNA stability via regulatory proteins, such as the Hu antigen R (HuR) (19). Other regulatory elements include GU-rich elements (GREs), CU-rich elements (CUREs), CA-rich elements (CAREs), iron responsive elements (IREs) and selenocysteine insertion sequence elements (reviewed in (20)).

**Figure 2.**
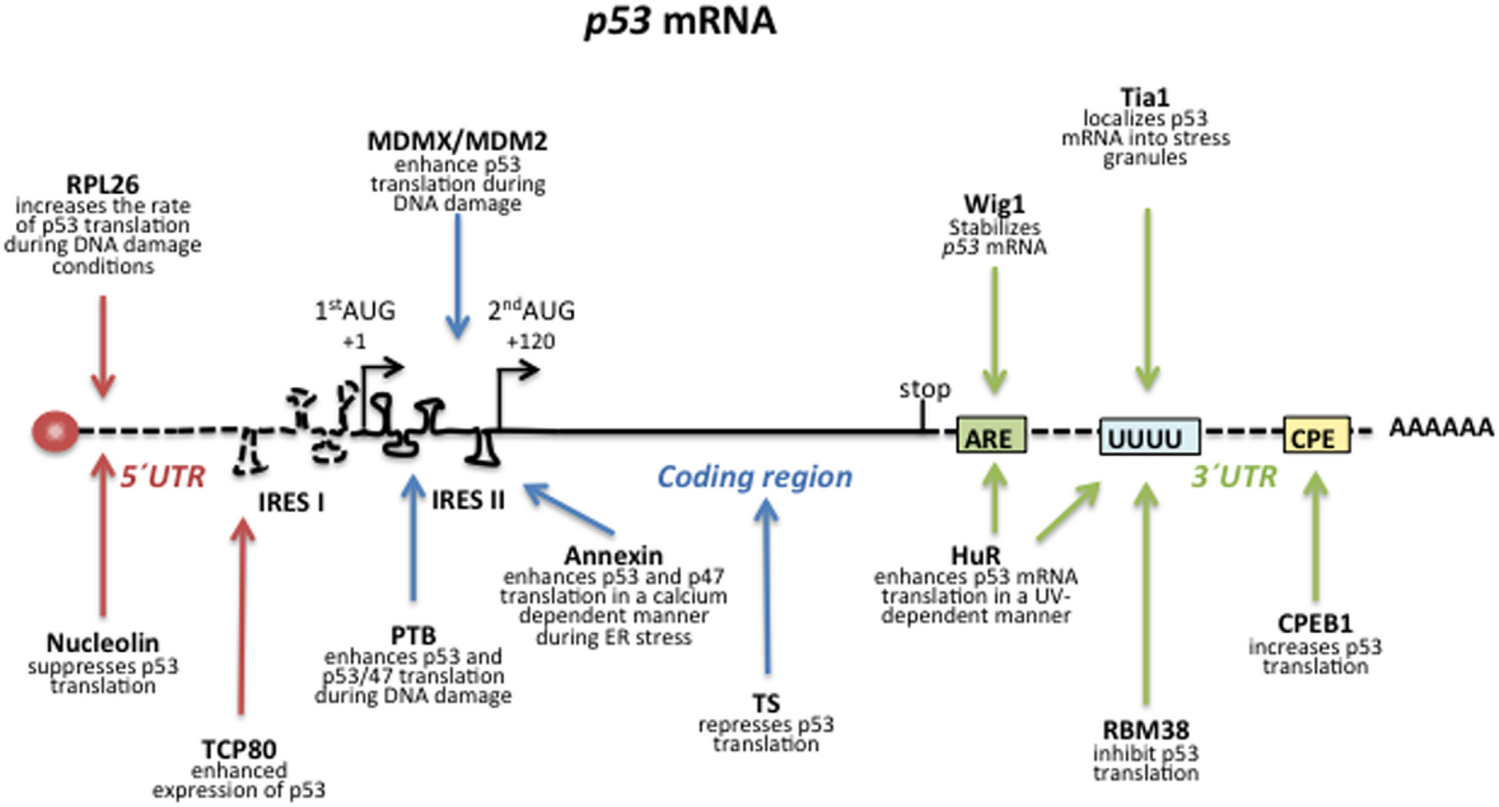
*p53 mRNA interacting factors*. In response to particular cellular conditions, a multitude of different proteins and RNA molecules interact with the *p53* mRNA to control p53 expression. The cartoon shows some *p53* mRNA binding factors. Please see also Tables 1, 3 and 4.

The *p53* mRNA 3′ UTR was recognized to play an important regulatory role in *p53* translation over 20 years ago by Fu *et al.*, who also confirmed that p53 protein levels did not correlate with *p53* mRNA levels in blast cells from patients with acute myeloid leukaemia (21). The role of the 3′ UTR in suppressing translation was further demonstrated in cell-free lysates using a chimeric reporter assay (21). Moreover, gamma irradiation relieved the 3′ UTR-mediated repression and resulted in an increased association of the *p53* mRNA with polysomes (9). In a later study, a *cis*-acting 66-nucleotide U-rich sequence was identified as a 3′ UTR repressive element together with an unknown associated 40 kDa protein (22). Since these early studies, several 3′UTR elements have been identified and these include AU-rich, U-rich and cytoplasmic polyadenylation signals that are recognized by binding factors (proteins, miRNAs, lncRNAs) and together participate in *p53* mRNA translation control in cell type- and condition-dependent responses (Table 1 and Figure 2).

**Table 1.** *p53* mRNA 3′UTR binding factors

Binding factor	Name	Binding sequence/region	Assay	Consequences	References
**HuR**	Human antigen R	AU-rich element/U-rich element	RNA EMSA, supershift, RNA-protein pulldown, IP of RNP complexes and RT-PCR, colocalization of HuR and p53 mRNA on cells polysomes, siRNA of HuR and impact on RNA stability, luciferase assay	HuR stabilizes *p53* mRNA and enhances *p53* mRNA translation in a UV- dependent manner and in VHL(+) renal carcinoma cells.	Mazan-Mamczarz 2003; Galbán 2003; Zou 2006; Tong 2009
**Hzf**	Hematopoietic zinc finger protein	AU-rich element/U-rich element	RNA EMSA, supershift, luciferase assay, IP of RNP complexes and PCR	p19Arf signalling leads to HuR and Hzf mediated *p53* mRNA translation	Nakamura 2011
**lnc RNA 7SL**	Long non-coding RNA 7SL	AU-rich element/U-rich element	RNA-protein pulldown, luciferase assay, colocalization on polysomes	lnc RNA 7SL represses *p53* mRNA translation by binding to AREs and competes with HuR	Abdelmohsen 2014
*miRNA-125b	-	AU-rich element/U-rich element	luciferase assay, RNA-Protein crosslink, colocalization on polysomes	miRNA 125b supresses translation following genotoxic stress. Interaction competed by HuR	Ahuja 2016
**Wig1**	Wild-type p53-induced gene 1	AU-rich element	luciferase assay, IP of RNP complexes and RT-PCR, RNA-protein pulldown	Wig-1 binds to and stabilizes *p53* mRNA through an AU-rich element (ARE)	Vilborg 2009
**PARN**	Poly(A)-specific ribonuclease	AU-rich element	RNA EMSA, supershift, RNA-protein pulldown, luciferase assay, IP of RNP complexes and RT-PCR,	PARN deadenylase destabilizing *p53* mRNA in non-stress conditions by	Devany 2013
**CPEB1**	Cytoplasmic polyadenylation element-binding protein 1	cytoplasmic polyA signal	IP of RNP complexes and RT-PCR	CPEB1 knockdown resulted in decreased *p53* mRNA translational.	Burns 2008; Burns 2011; Glahder 2011
**RBM38 (RNPC1)**	RNA binding motif protein 38	U -rich element	RNA EMSA, IP of RNP complexes and RT-PCR	RBM38 binds to 5′ and 3′ UTR and inhibits translation. RBM38 upon phosphorylation at Ser 195 turns from being repressor to activator of p53 translation. RBM38 is dephosphorylated by PPM1D phosphatase resulting in p53 translational repression	Zhang 2011; Zhang 2013; Zhang 2015
**RBM24**	RNA binding motif protein 24	U -rich element	RNA EMSA, IP of RNP complexes and RT-PCR, luciferase assay	RBM24 repress p53 translation by prevnting the interaction with eIF4E.	Zhang 2018
**Tia1**	Cytotoxic granule associated RNA binding protein	U -rich element	iCLIP, luciferase assay	Tia1 targets *p53* mRNA to stress granules under normal, but not stress conditions.	Díaz-Muñoz 2017
**40 kDa unknown protein**	-	U-rich element	Luciferase assay, RNA-Protein crosslink	-	Fu 1999

*For review of miRNA see Liu *et al.* 2017.

The AU-rich binding protein HuR was identified by Mazan-Mamczarz *et al.* as a direct binding partner of the *p53* 3′ UTR following UV-C irradiation (23). Despite a reported role of HuR increasing the stability of several transcripts (24–26), changes in *p53* mRNA abundance or stability were not observed and only an enhanced translational rate was reported. However, an HuR-dependent *p53* mRNA stabilization and greater p53 protein levels were reported in renal carcinoma cells expressing the Von-Hippel-Lindau (VHL) tumour suppressor, as compared to VHL (-) cells (27). Moreover, HuR was shown to play a role in flavonoid apigenin-induced p53 protein synthesis in keratinocytes (28). Findings from Nakamura *et al.* revealed that HuR acts on the *p53* mRNA in cooperation with Hzf1 and that these proteins are necessary for p19ARF-induced p53 expression in mice (29). Two RNAs have been described to compete for binding with HuR and to suppress p53 synthesis. The noncoding 7SL RNA is upregulated in some cancers and can form a partial hybrid with the 3′ UTR region of the *p53* mRNA and silencing of 7SL results in an increased HuR - *p53* mRNA interaction and enhanced translation (30). A similar RNA-dependent suppression of p53 synthesis was reported by the genotoxic-induced miR-125b. Single nucleotide variations (SNV) have been identified in the *p53* 3′UTR of patients with diffuse large B-cell lymphoma, three of these disrupt the match between miR-125 - *p53* mRNA inhibiting suppression of p53 (31) (Table 2). The *p53* mRNA-miR-125 interaction was also competed by HuR binding to the U-rich element close to the miR-125b target site, dissociating the *p53* mRNA from the RISC complex and preventing its degradation (32). The HuR is known to translocate to the cytoplasm upon several stress conditions and to impact the synthesis of several proteins but it is not clear if this translocation is important for controlling p53 expression.

**Table 2.** Diseases associated synonymous mutation on p53

Region	Disease	Interacting factor	Type	Physiological consequences/cellular conditions	References
**CDS**	Colorectal carcinomas	MDM2	Mutation	V10V (GTC>GTT) silent mutation with reduces affinity for MDM2, then with lower p53 expression levels.	Hayes 1999; Candeias *et al.* 2008
**CDS**	Chronic lymphocytic leukemia	MDM2	Mutation	L22L (CUA> CUG) silent mutation impair Mdm2-mediated enhancement of *p53* translation	Oscier 2002
**CDS**	Nonmelanoma skin cancer	MDM2	Mutation	P36P (CCG>CCT) silent mutations has a reduce affinity for MDM2	Candeias 2008; Kanjilal 1995
**5**′**UTR**	Melanoma	PTB	SNP	SNP at position 119 in the 5′ UTR of *p53* mRNA has consequences on the translational control of p53. This may relate with a weaker IRES activity	Khan 2013
**3**′**UTR**	Diffuse large B-cell lymphoma (DLBCL)	miR125-b	SNV	Three SNVs disrupt the interaction between miR-125b and the *p53* 3′UTR, thereby impeding suppression of p53.	Li 2013

Other *p53* mRNA ARE-binding proteins include Wig1 (wild-type p53-induced gene 1) which binds to AREs of several RNAs important in cell cycle regulation such as *FAS* (33,34). A feed-back mechanism has been suggested by which Wig1 transcription is regulated by p53 and where Wig1 protects the *p53* mRNA from deadenylation (35,36). The PARN (poly(A) specific ribonuclease) also interacts with the *p53* ARE but instead promotes deadenylation and is suggested to keep p53 levels low in non-stressed conditions (37). The activity of PARN is regulated via adjacent ARE sites that bind miR-504 and miR-125b (38). The effect of PARN on the *p53* mRNA can also be indirect and it was shown that knock-down of PARN in gastric cell lines results in an increase in p53 protein levels but without the expected destabilizing effect on the *p53* mRNA (39).

The *p53* mRNA 3′ UTR contains two highly conserved U-rich sequences belonging to cytoplasmic polyadenylation elements (CPEs) that are located upstream of the polyadenylation sequence and play a role, as the name indicates, in the regulation of poly (A) tail length in the cytoplasm. Rosentierne *et al.* showed that CPEs influence the stability and the translational rate of the *p53* mRNA (40) and by using wild type and mutant p53 sequences together with SILAC they identified several UV irradiation-dependent CPE-interacting proteins (CPEBs), such as the hnRNP1 and AUF1 (41). Later studies have identified additional *p53* mRNA CPEs binding proteins. CPEB1 acts in cooperation with other factors to regulate translation of several mRNAs, resulting either in activation or repression (42). Burns and Richeter showed that CPEB1 knockdown cells exhibit a general decrease in translational efficiency with an overall impact on the regulation of metabolic processes and senescence (43). The same group later observed a CPEB1-dependent shortening of the *p53* mRNA poly(A) tail and defined germline development 2 and 4 (Gld2 and Gld4) as two poly(A) polymerases acting together with CPEB1 on *p53* mRNA translation regulation. Surprisingly, depletion of Gld2 increased p53 polyadenylation and this effect was attributed to miR-122 binding sites in the *CBEP1* 3′ UTR. Gld4, on the other hand, acts together with CBEP1 to promote *p53* mRNA polyadenylation (44). More work is needed to better understand Gld2-mediated suppression of CPEB1 but, nevertheless, this raises interesting questions regarding the role of different cytoplasmic poly(A) polymerases acting on different target messages with different outcomes (Table 1). The involvement of Gld family of proteins in p53 regulation was also observed in *C. elegans*, where the association of Gld1 with 3′ UTR of *CEP-1* (the only *C. elegans* p53 family member) leads to repression of translation of *CEP-1* and regulates the induction of apoptosis after DNA-damage during meiosis (45).

Proteins from the RBM family (RNA binding motif family) are also involved in controlling *p53* mRNA translation via the 3′ and/or 5′ UTRs. RBM38 (also called RNPC1), is known to be overexpressed in canine lymphomas and was shown to bind to a U-rich element in the 3′ UTR or to the 5′ UTR and to repress *p53* translation by preventing eIF4E from binding the p53 message via a direct interaction (46). This repression can be abolished by the GSK3-dependent phosphorylation of RBM38 at serine 195 and results in the activation of *p53* translation (47). RBM38 is a target gene for the p53 family, suggesting a feedback to control p53 synthesis under normal conditions. Furthermore, p53 induces the PPM1D phosphatase that dephosphorylates RBM38 on serine 195 (48). An RBM38 deficient mouse model showed the importance of RBM38 in haematopoiesis and p53-mediated radio sensitivity and tumour suppression (49). RBM24 shares a high level of homology with RBM38 and acts in a similar fashion to repress p53 synthesis. RBM24 deficient mice displayed heart developmental failure, resulting in embryonic lethality that was partially rescued by p53 deficiency. Both RBM24 and RBM38 proteins represent interesting pathways for tissue-specific regulation of p53 synthesis. However, more *in vivo* experiments are needed to determine to what extent the *p53* mRNA interaction with these proteins is responsible for the reported phenotypes. An interesting aspect is that RBM38 also binds the AU/U-rich elements in the 3′ UTR of p63, another p53 family protein, and inhibits the synthesis of p63 indicating a putative co-regulation of p53 and p63 (50).

Tia1 is an RNA-binding protein playing a role in the formation of stress granules, which are complexes of RNA binding proteins and translationally silenced mRNAs. Stress granules are assembled by different proteins and different pools of mRNAs depending on stress type and are connected to diseases such as neurodegenerative diseases and cancer (51). B-lymphocytes treated with the DNA-damaging agent etoposide showed a dissociation of *p53* mRNA from stress granules leading to p53 activation, that is mediated by Tia1 and an U-rich sequence of the *p53* mRNA (52). The role of *p53* mRNA in stress granules is worthwhile to study in more detail during different stress conditions. In addition to direct protein binding elements, a G-quadruplex RNA structure downstream of the poly(A) signal was identified to play a role in pre-mRNA splicing during DNA damaging conditions together with hnRNP H/F and RNA helicase DHX36 (53,54).

More than 20 microRNAs that are elevated in tumours have been reported to target the 3′ UTR, such as miR-504 (55), miR-33 (56), miR-98, miR-380-5p (57) (for review see (58)). miR-1285 shows some overlapping binding regions with the Polypyrimidine Tract Binding Protein (PTB) in the *p53* 3′UTR. knockdown of PTB leads to a decrease in translation of the p53 and Δ40p53α isoforms through the 3′UTR. A knockdown of miR-1285, enhance the association of PTB with the 3′UTR. The interplay between miR-1285, *p53* 3′UTR and PTB play a pivotal role in the expression of both isoforms of p53 (59). Some other regulatory RNAs (long non-coding RNAs) also impact *p53* translation via direct or indirect interactions (for review see (60)).

### The role of the *p53* mRNA 5′UTR

The ∼140 nucleotide *p53 mRNA* 5′ UTR also plays an important role in controlling translation efficiency. An early study showed that the full-length p53 selectively binds its own 5′ UTR and inhibits translation (61). Since then, several studies have identified the *p53* 5′ UTR as a major site for controlling stress responsive *p53* mRNA translation. Takagi and colleagues showed that the ribosomal protein RPL26 and nucleolin act as modulators of *p53* translation both *in vitro* and in cell-based studies (62). RPL26 preferentially binds to the *p53* 5′ UTR and enhances translation in response to genotoxic stress whereas nucleolin suppresses translation under normal conditions (Figure 2). A model was proposed in which nucleolin inhibits *p53* translation to maintain p53 expression at low levels under normal conditions while after cellular stress nucleolin competes with RPL26 to enhance p53 synthesis. Interestingly, a dsRNA sequence that links the 5′ UTR with the 3′ UTR sequences was shown to be critical for RPL26-mediated translation regulation (63,64).

mRNA translation is predominantly initiated via the 5′ m^7^G cap structure to which eIF4E binds followed by the recruitment of eIF4A and eIF4G and the formation of the 43S pre-initiation complex and the assembly of 80S at the first in frame downstream AUG. However, some mRNAs bypass the need of the cap structure and recruit the 40S ribosomal subunit directly to the mRNA via so-called Internal Ribosome Entry Sites (IRESs). The underlying molecular mechanisms for cap-independent translation are best described for viral mRNAs (HCV, HIV, etc.) but also cellular mRNAs can be initiated using IRESs. These RNA elements are normally located in the 5′ UTRs and have precise tertiary structures to allow a selective interaction with so-called IRES trans-acting factors (ITAFs) and ribosome components (65,66). In 2006, some groups reported IRES-like activity on the *p53* 5′ UTR (IRES I) (67,68). Several RNA-binding proteins have since been identified that interact with this region (Table 3). PTB also binds the 5′UTR and positively acts as an ITAF and regulates IRES activity by relocating from nucleus to the cytoplasm during stress conditions (69). *In vitro* PTB binding and *ex vivo* PTB-knockdown studies have shown that a single-nucleotide polymorphisms (SNPs) C119T in the *p53* 5′ UTR prevents PTB binding and hence is refractory to PTB knockdown (Table 2). This provided the first evidence that mutations or polymorphism in the 5′ UTR of *p53* can influence translational regulation (70). Programmed cell death protein 4 (Pdcd4), was proposed to act as a translational suppressor of *p53* mRNAs by directly interacting with translation initiation factor eIF4A to help maintain a low level of p53 synthesis in unstressed cells (71). This suppression is abrogated due to a decrease in Pdcd4 following DNA damage.

**Table 3.** *p53* mRNA 5′UTR binding factors

Binding factor	Name	Binding sequence/region	Assay	Consequences	References
**p53**	Cellular tumor antigen p53	–216 to +1	RNA-protein pull down	p53 binds to the 5′ UTR region and inhibits its own mRNA translation.	Mosner 1995
**L26 (RPL26)**	Ribosomal protein L26	–191 to 2	RNA EMSA; IP-RT-PCR	RPL26 preferentially binds to the 5′ UTR after DNA damage and enhances translation.	Takagi 2005
**Nucleolin**	Nucleolin	–191 to 2	RNA EMSA; IP-RT-PCR	Nucleolin overexpression suppresses p53 translation	Takagi 2005
**Wrap53**	WD repeat containing antisense to TP53	IRES I	Luciferase assays; RNase protection assay; RNA-ChIP	Overexpression of Wrap53 increases *p53* mRNA and protein levels.	Mahmoudi 2009
**RBM38 (RNPC1)**	RNA binding motif protein 38	IRES I	RNA EMSA; RT-PCR; Quantitative PCR; RNA-ChIP	RNPC1 inhibits expression of p53 via *p53* 5′ or 3′ UTR.	Zhang 2011
**Pdcd4**	Programmed cell death 4	IRES I	IP-RT–PCR	The translation inhibitory effect of Pdcd4 is mediated by the 5′UTR and depends on the ability to interact with eIF4A.	Wedeken 2011
**hnRNP Q**	Heterogeneous nuclear ribonucleoprotein Q	IRES I	UV cross-linking followed by Immunoprecipitation (CLIP)	hnRNP Q binds to the 5′UTR of mouse *p53* mRNA and regulates translation efficiency of p53 and apoptosis progression.	Kim 2013
PTB**	Polypyrimidine tract-binding protein	IRES I	UV cross-linking followed by Immunoprecipitation (CLIP)	PTB is an IRES trans-acting factor that positively regulates p53 IRES-I activity.	Khan 2013
**DAP5**	Death associate protein 5 (also NAT1 or p97)	IRES I	RNA-protein crosslink; Bicistronic constructs; Luciferase assay; IP of RNP complexes and RT-PCR	Positively regulate the translation of various IRES containing mRNAs, promotes IRES-driven translation including the *p53* mRNA.	Weingasten-Gabbay 2014
**TCP80**	Translational control protein 80	IRES I	Dual-Luciferase assays; IP-RT-PCR	Overexpression of TCP80, together with RHA, leads to enhanced p53 expression.	Halaby 2015
**RHA**	RNA helicase A	IRES I	Dual-Luciferase Assays; IP-RT-PCR	Enhances *p53* mRNA translation.	Halaby 2015
**Ku**	Ku	IRES I	IP-RT-PCR ; RNA EMSA; UV cross-linking	Ku represses p53 protein synthesis and p53-mediated apoptosis by binding to a bulged stem-loop structure within the 5′ UTR.	Lamaa 2016
**hnRNP L**	Heterogeneous nuclear ribonucleoprotein L	IRES I	IP-RT-PCR	hnRNP L binds the 5′UTR. Knockout of hnRNP L decrease of p53 levels	Seo 2017

**PTB also interacts with the 3′UTR (Katoch *et al.* 2017).

The heterogeneous nuclear ribonucleoprotein Q (hnRNP Q) was shown to bind to the 5′ UTR of the *Per1* message and control rhythmic cap-independent translation in mammalian circadian system. It was reported, using a luciferase reporter system, that under normal and stress conditions, hnRNP Q affects the cap-independent translation efficiency of the mouse *p53* mRNA (72).

In 2015 Halaby *et al.*, shown that the Translation Control Protein 80 (TCP80/NF90) interacts with the *p53* 5′ UTR throughout immunoprecipitation assays. They have also shown that TCP80 is associated with RNA helicase A (RHA) following DNA damage and this association has a positive effect on the *p53* IRES activity (73). TCP80/NF90 has also been identified to interact with the acid-beta glucosidase mRNA and to suppress translation (74). Ku is known as a main sensor and repair factor of double-strand DNA breaks (DSBs) and was shown to interact with a stem-loop structure within the *p53* 5′ UTR. Like with Pdcd4 and RBM38, Ku contributes to maintaining low p53 levels in cells under normal conditions by suppressing translation. Ku's inhibitory mechanism is abrogated during DNA damage via Ku acetylation (75). Dimers of Ku70/80 were previously shown to bind and stimulate translation of IRESs-carrying messages such as the *VEGF* and *PDGF*. Hence, both the TCP80/NF90 and Ku have reported opposite effects on translation control, depending on the message and cellular conditions. For DNA damage sensing and repair, Ku acts together with DNA-PK but it is not clear if this complex is also acting on translation. Recently, heterogeneous nuclear ribonucleoprotein L (hnRNP L) was predicted as a *p53* mRNA binding factor regulating p53 levels under cell stress conditions. Further experimental analyses indicate that hnRNP L associates with the 5′ UTR of *p53* mRNA serving as an ITAF. Knock-down of hnRNP L led to a decrease in p53 protein levels, suggesting that hnRNP L acts as a positive regulator of *p53* translation and can promote apoptosis and cell cycle arrest following DNA damage (76). Not only proteins have been shown to target the *p53* 5′ UTR but also the long non-coding natural antisense transcript of p53 (Wrap53). Computational analyses and experimental results suggest that *Wrap53* is a highly conserved natural antisense transcript that regulates *p53* mRNA levels (77–79). Taken together, a number of studies have identified factors interacting with the 3′ and the 5′ UTRs of the *p53* message and controlling its rate of translation under both stressed and unstressed conditions. It will be interesting to explore how these different factors act together, or independently, to control p53 synthesis in response to different signalling pathways.

### The role of the *p53* mRNA coding sequence

The role of coding sequences in controlling translation is less studied but it is well known from dendrite cells, or the spatial expression of some bud-tip mRNA in yeast, that proteins binding to the coding regions regulate mRNA transport and spatio-temporal protein expression (for review see (80)). The p53/47 isoform is derived from alternative initiation of translation at the second in frame AUG codon via a second IRES (IRES II) located inside the coding region within the first 120 nucleotides (+1 to +120) downstream of the 1st AUG (68,69,81) (Figure 2). Hence, despite being within the coding region of the full-length p53, IRES II constitutes the 5′ UTR of the p53/47. Some factors have been shown to interact with this region, including the polypyrimidine-tract-binding protein (PTB) that enhances synthesis of p53 and p53/47 in response to genotoxic stress. (82) (Table 4). The binding of PTB associated Splicing Factor (PSF/SFPQ), or Annexin A2, to the 5′ of the coding sequence (+1 to +251) stimulates synthesis of both isoforms. However, PSF and Annexin A2 compete for RNA binding, suggesting a similar effect on *p53* translation but via different signalling pathways. For example, the binding of Annexin A2 is calcium-dependent and an increase in Annexin A2 - *p53* mRNA interaction was shown following thapsigargin treatment (83). The Death Associate Protein 5 (DAP5), also named p97 or NAT1, is a member of the translation initiation factor 4G (eIF4G) family (84) and binds the *p53* coding sequence and enhances the synthesis of the p53/47 isoform and to a less extent full-length p53. DAP5 has the potential to bind both IRES elements but it preferentially promotes initiation from the second AUG governed by IRES II (85). These different reports suggest that factors binding to the 5′ UTR or the coding sequence of p53 can differentiate the synthesis of p53 full length and/or p53/47 in response to different cellular conditions (Figure 2).

**Table 4.** *p53* mRNA coding region binding factors

Binding factor	Name	Binding sequence/region	Assay	Physiological consequences/cellular conditions	References
**DAP5**	Death associate protein 5 (also NAT1 or p97)	IRES I and II	RNA-protein crosslink; Bicistronic constructs; Luciferase assay; IP of RNP complexes and RT-PCR	Enhances p53/47 expression and to a lesser extend p53 full length.	Weingasten-Gabbay 2014
**PTB**	Polypyrimidine-tract-binding protein	–1 to +39 (IRES II)	Bicistronic constructs; Luciferase assay; Filter binding assay	Enhances p53 and p53/47 translation following genotoxic stress.	Grover 2008
**PSF/SFPQ**	PTB associated Splicing Factor	+1 to +251	RNA affinity chromatography (RNA-protein pull-down), IP of RNP complexes and RT-PCR; Filter binding assay	Enhance p53 and p53/47 expression.	Sharathchandra 2012
**Annexin A2**	Annexin A2	+1 to +251	RNA affinity chromatography (RNA-protein pull-down), IP of RNP complexes and RT-PCR; Filter binding assay	Enhances p53 and p53/47 translation in a Ca^2+^ dependent fashion after thapsigargin- induced ER stress.	Sharathchandra 2012
**MDMX (MDM4)**	Murine double minute 4	IRES II	RNA-IP: RNA ELISA	Chaperoning *p53* mRNA to provide an MDM2 binding platform following genotoxic stress.	Malbert-Colas 2014
**MDM2**	Murine double minute 2	IRES II	RNA-IP; RNA EMSA; RNA-ELISA	Enhance p53 translation during genotoxic stress.	Candeias 2008, Gajjar 2012
**APP**	Amyloid precursor protein	+1 to +120	RNA-IP; RNA-protein pull down; Luciferase assay; Bicistronic constructs	Enhances p53/47 expression.	Li 2015
**TS**	Thymidylate synthase	+531 to +1020	IP of RNP complexes and RT-PCR; In vitro translation assay; RNA EMSA	Represses translation. The levels of *p53* mRNA increase in RNP complexes.	Chu 1999

The thymidylate synthase (TS) interacts with the *p53* mRNA further towards the 3′ in a region comprising 489 nt between +531 to +1020, resulting in repression of *p53* translation and the association of the *p53* mRNA with a ribonucleoprotein complex (RNP) (86,87). MDM2 and MDMX also bind (+1 to +120) and control p53 synthesis and this will be discussed in detail in the ‘*riboswitch-like behaviour*’ section.

### Physiological aspects of alternative initiated isoforms

Activation of the unfolded protein response (UPR) constitutes a physiological pathway for inducing p53/47 expression (88). The UPR is triggered by an accumulation of misfolded proteins in the endoplasmic reticulum in response to both intra- and extra-cellular stresses such as high protein synthesis or oxidative stress, or poor tissue perfusion with low oxygen and nutrient levels (89,90). The cellular response aims to restore the balance of mature versus newly synthesized proteins via three different effector pathways (IRE-1, ATF-6 and the PERK kinase) that originate in the BiP (also known as GRP-78 or HSPA5) chaperone binding to misfolded protein in the ER lumen and thereby releasing the effector proteins. PERK is a general inhibitor of cap-dependent mRNA translation via phosphorylation of eIF2α. But some mRNAs that are important for ER repair such as ATF4, are instead activated by PERK (91). The *p53* mRNA is a PERK-response message and suppression of PERK activity prevents ER stress-induced expression of p53/47 (88). Interestingly, the PERK response sequence in the *p53* mRNA also confers an increase of full-length p53 following DNA damage (see further above and below). Hence, the same RNA sequence plays different roles on controlling p53 isoform synthesis depending on the signalling pathway.

The p53/47 lacks the first 40 amino acids including the first transactivation domain (TA I) and the MDM2 binding site (MDM2 is a key regulator of p53 that binds both the *p53* mRNA and protein, see further below) and therefore p53/47 has different activity and stability as compared to full-length p53. However, p53/47 retains the DNA binding and oligomerization domains and adds further levels of regulation of p53 activity as homo- or heterodimers. As a homo-tetramer, p53/47 induces G2 cell cycle arrest but has no effect on progression through the G1 phase of the cell cycle, while the opposite is the case for full length p53 that causes G1 arrest but has little effect on the G2 (88). The key to the different effects of these two isoforms on cell cycle progression is the cell cycle kinase inhibitor p21/waf1^CDKN1A^. Full-length p53 induces p21/waf1^CDKN1A^ and G1 cell cycle arrest following DNA damage whereas p53/47 actively suppresses p21/waf1^CDKN1A^ expression due to the fact that it lacks TA I plus that it actively suppresses p21/waf1^CDKN1A^ synthesis (92). The suppression of p21/waf1^CDKN1A^ causes a p53/47-dependent induction of 14–3-3σ and G2 cell cycle arrest via sequestering CDK1. As there is an estimated 30% less protein synthesis during the G2 phase of the cell cycle it is thus preferable for the cells to arrest in G2 to carry our ER repair (93). G1 arrest triggered by full-length p53-induced p21 expression allows cells to repair DNA damage before replicating the DNA. The UPR pathway is dominant and cells where both the DNA damage and UPR pathways are activated induce p53/47 and fail to arrest in G1. Such activation of both pathways is reminiscent of poorly perfused tumours with unstable DNA or undergoing genotoxic treatment (92). Prolonged ER stress results in p53/47-dependent apoptosis that is not accompanied with the induction of the pro-apoptotic factors that are characteristic for full-length p53-induced apoptosis. Instead, p53/47-induced apoptosis is mediated by suppression of BiP synthesis that results in the displacement of BIK (Bcl-2 induced killer) from BiP (94). It is interesting that both p53/47-dependent cell cycle arrest and apoptosis depend on suppression of translation of specific mRNAs.

Animal studies have shown that overexpression of p53/47 (p44 in mice) in a p53wt background results in a pre-mature ageing phenotype via hyper activation of IFG-1R (95). This indicates that a balanced expression of the two isoforms is required under physiological conditions to maintain tissue homeostasis and has also led to the suggest that p53/47 is associated with longevity (96). Later works showed a cognitive impairment associated with altered activity of tau via a p53/47-dependent regulation of several tau kinases, such as GSK3b, Dyrk1a and Cdk5. The phenotype could be rescued by *Igfr1* or *Mapt (tau)* haploinsufficiency (96). The hyperphosphorylation of tau has been linked with age-associated tauopathies as Alzheimer diseases (96–98). In this context it is also interesting that the amyloid precursor protein (APP) C-terminal domain controls p53/47 expression, providing a potential link between p53/47, ageing and neurodegenerative disease (93) (ref).

### Riboswitch-like behaviour of the *p53* mRNA

During the DNA damage response, the +1 to +120 coding sequence of the *p53* mRNA shows conceptual similarities with prokaryotic riboswitches. Riboswitches serve to quickly adapt protein expression to changes in the cellular environment. They consist of two RNA structures that are usually located in 5′ UTRs. A ligand, often a metabolite, binds the aptamer that controls the expression platform and determines the rate of translation of an enzyme that acts in the corresponding metabolic pathway (99–104). The molecular mechanism of this on-off like mechanism is based on ligand-dependent changes in RNA structures (105). Relatively little is known regarding how this adaption of gene expression to changes in the cellular environment has evolved in higher eukaryotes but the induction of p53 synthesis by the E3 ubiquitin ligase MDM2 following DNA damage shows some interesting parallels. The *p53* mRNA–MDM2 interaction is regulated by the ATM kinase via phosphorylation of MDM2 at serine 395 that opens MDM2’s RNA-binding pocket to bind an RNA structure within the (+1 to +120) sequence (106). But the interaction also depends on the folding of the *p53* mRNA and this is also regulated by ATM but via phosphorylation of the MDM2 homologue, MDMX at serine 403. MDMX acts as an RNA chaperone to form the platform to which MDM2 can bind. The platform consists of three stem loop structures (SL I, II and III). The central hairpin (SL-II) is the highly conserved *box-I* that under normal conditions is associated with SL-I via an interaction between codons 10 and 21. In the presence of MDMX, SL-II instead forms an interaction between codon 22 of SL-II and codon 41 of SL-III to form the MDM2 binding platform (107). The synonymous cancer-derived mutation CUA to CUG (codon leucine 22) that is located within the conserved *box-I*, obstructs the folding of the RNA and prevents MDM2 binding and the induction of p53 expression after genotoxic stress (108). It is, perhaps, surprising that a single nucleotide can have such an important effect on an RNA structure but it is plausible that this reflects the fact that *in vivo* regulated RNA structures need to be flexible.

### The evolution of the *p53* box-I interaction with MDM2

The interaction between the *box-I p53* mRNA and MDM2 is also present in the pre-vertebrate *Ciona instestinalis* (109). The pre-vertebrate *p53* RNA structure is regulated by temperature and the optimal binding temperature for the *p53* mRNA–MDM2 protein interaction for *C. intestinalis* was measured at 18 and 30°C for the mammalian (107–109). Temperature-dependent RNA folding, or so-called RNA thermometers, is a common feature affecting differentiation processes during embryogenesis and development of various organisms and in viruses (110), bacteria (111), yeast (112), corals (113) and plants (114). Computational prediction backed up by experimental data show structural similarities when simulating the temperature of the natural environment of different species (including Protozoa, Nematoda, Cnidaria and Tunicata) (115–118). Comparative studies on *C. intestinalis* and mammalian *p53* mRNA – MDM2 interactions indicate that the folding of the *p53* mRNA and the stress-responsive expression of p53 has evolved from a temperature-induced sensing mechanism to a signaling pathway regulated RNA-chaperone mechanism. Interestingly, some well-studied developmental models that lack MDM2/MDMX express several p53 isoforms. In *Drosophila melanogaster* there are four *predicted p53* mRNA isoforms (A, B, C and E) and three proteins, with transcript A and C encoding the same 44 kDa protein (119,120). In *C.elegans*, the regulation of *cep-1/p53* translation was shown to promote DNA damage-induced apoptosis demonstrating the physiological role of this mechanism (45).

The *box-I* RNA sequence encodes the peptide domain required for the docking of MDM2 to the p53 protein that precedes MDM2-dependent degradation of p53 under normal conditions. Hence, from the same DNA sequence have evolved two MDM2-binding motifs: one RNA structure controlling the rate of synthesis and one peptide domain controlling the rate of degradation. This raised the question which of these two functions of MDM2 evolved first. Peptides (20 amino acid long) corresponding to the BOX-I of human (Hu-p53) and *C. intestinalis* p53 (Ci-p53) were shown to bind both human and *C. intestinalis* MDM2 (Hu-MDM2 and Ci-MDM2). However, full-length Ci-p53 protein does not bind Hu-MDM2. Interestingly, the interaction was restored by deleting a non-conserved flanking region of Ci-p53 BOX-I. As the *p53* mRNA–MDM2 interaction is present in *C. intestinalis*, this suggests that the *p53* mRNA–MDM2 interaction preceded the p53–MDM2 protein–protein interaction and that the latter evolved by elimination of the BOX-I flanking region.

There are interesting evolutionary aspects related to RNA sequences within the coding sequence and the function of the encoded protein. During the eukaryotic evolution, there is a prominent correlation between transcription factors contributing to the origins of multi-cellularity and embryonic development along with the increasing complexity of the organisms (121,122). In higher organisms, classic structural proteins are mainly involved in metabolic processes, while disordered proteins play roles in cellular signaling and molecular recognition pathways. The latter require high specificity along with low affinity to confer dynamic folding and interactions exchanges (123–125). The p53 *box-1* sequence is an interesting example of how an RNA structure has evolved from an RNA thermometer to a chaperone-regulated riboswitch in mammalian cells that respond to DNA damage and ER stress pathways while at the same time the encoded peptide domain has evolved to the TA I domain that gives p53 a multifunctional gene target regulatory capacity. Some good examples of structured RNA evolution come from viruses. For example, the secondary structure of the human immunodeficiency virus 1 (HIV-1) genome suggests a reciprocal interference between selection at the RNA and protein levels, by which certain RNA structures are maintained in evolution (126). The molecular basis for HIV-1 evolution and subtype differentiation is based on a high mutation rate (127) and specific RNA motifs have been proposed to drive the mutagenesis in response to various factors, including phenotypic selection and exogenous selection pressure, such as antiviral drugs (128).

A fundamental characteristic of the secondary RNA structure is the highly conserved G·U wobble base pairs which have unique chemical, thermodynamic and structural properties (129). Such pairs allow conformational flexibility and are conserved in the acceptor helix of tRNA of Ala (130) and on mRNAs encoding ribosomal proteins (131–133) and are functionally associated with self-splicing ribozymes (i.e. Hepatitis delta virus) (134,135).

These different studies highlight that phenotypic diversification among species should include both RNA and peptide sequences and structures (136).

### The *p53* mRNA as a protein ligand

**Figure 3.**
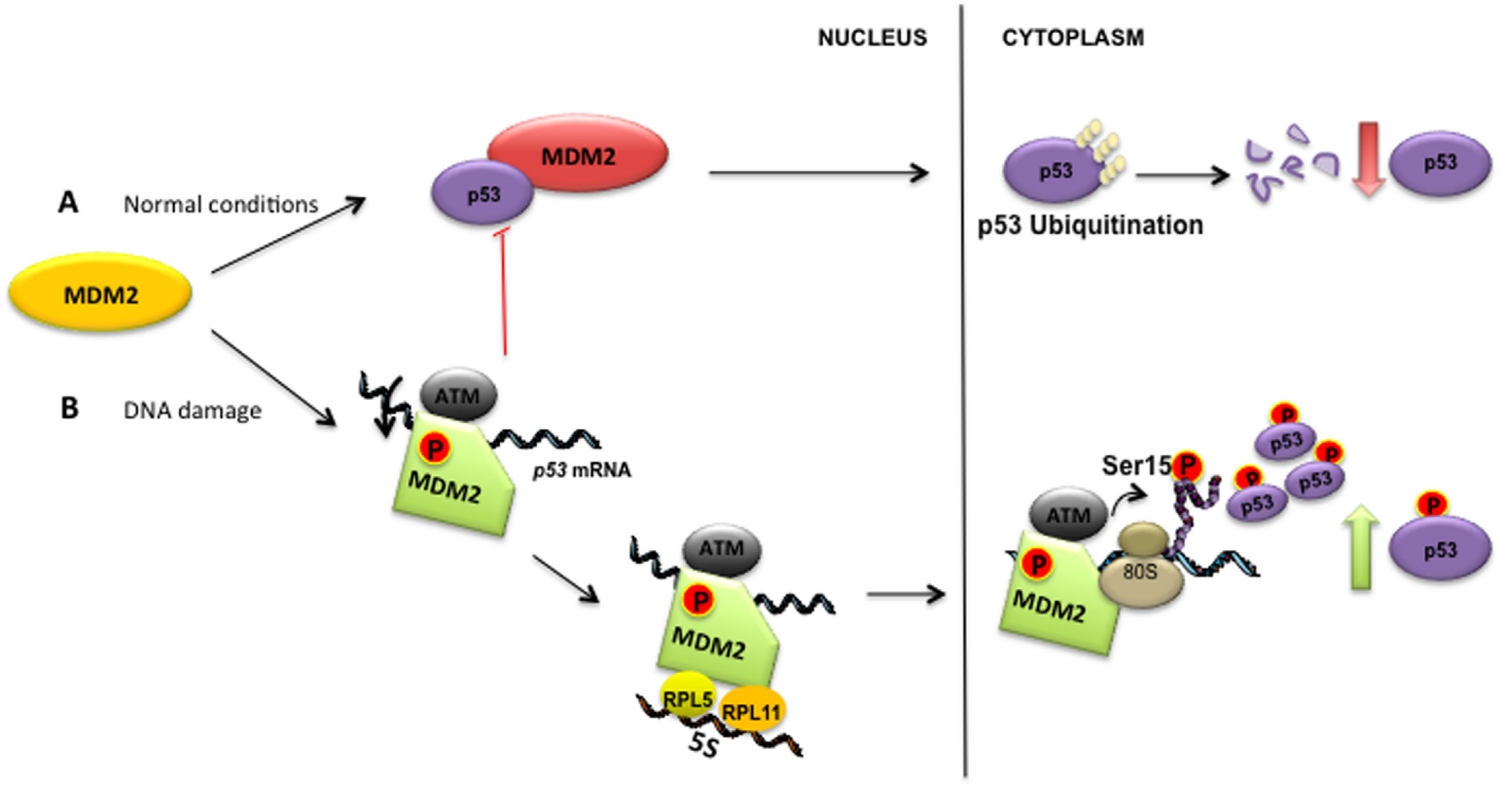
*MDM2’s switch from a negative to a positive regulator of p53 following DNA damage is dependent on the p53 mRNA*. (**A**) Under normal conditions, MDM2 (in red) binds and catalyzes the ubiquitination of p53, targeting it for degradation via the 26S proteasomal pathway (of note, p53 degradation can also take place in the nucleus (165)). (**B**) Following DNA damage, ATM phosphorylates MDM2 at Ser395 (in green) that allows MDM2 to interact with the *p53* mRNA. This promotes the interaction with the 5S RNP complex and an increase in p53 synthesis. The *p53* mRNA–MDM2 is required to bring ATM to the p53 polysome where it phosphorylates the nascent p53 protein to prevent MDM2-mediated degradation of the newly synthesized p53. A single synonymous mutation in the *p53* mRNA at codon 22 prevents MDM2 from binding the *p53* mRNA and prevents the stabilization of p53 following genotoxic stress.

Controlling p53 expression is critical for cell homeostasis and too little activity can pave the way for tumour development whereas too much activity can cause cell death. MDMX helps MDM2 to fine-tune MDM2’s activity towards p53, both in terms of synthesis and degradation, but it lacks the capacity to affect p53 expression on its own, despite a 75% sequence similarity between their respective RING domains (137–140). Both MDM2 and MDMX are essential during development and knock out of either factor is lethal in mice during early embryogenesis. However, animals that also lack p53 develop normally (141,142). MDM2 is an early p53 target gene, which implies a p53-MDM2 feedback loop. This physiological role of this feedback loop was tested by mutating the p53 binding site of the *mdm2* promoter and it was shown that these animals developed normally and that p53-mediated control of MDM2 expression is not required during development. However, these animals showed an impaired DNA damage response, suggesting that a positive p53-MDM2 feedback loop is important during the DNA damage response to activates p53 (143). MDM2’s switch from a negative to a positive regulator of p53 following DNA damage starts with the activation of the ATM kinase that phosphorylates MDM2’s RING domain at serine 395 (394 in mouse) (as discussed above) (144). This event does not inhibit MDM2 E3 ubiquitin ligase activity and the phosphomimetic MDM2S395D mutant exhibits E3 ligase activity towards p53. However, the binding of the *p53* mRNA to the C-terminus of MDM2S395D prevents MDM2 from targeting p53 for degradation by inducing allosteric modifications in the N-terminus of MDM2 that prevent its interaction with the p53 protein (106,140,145) (Figure 3). The binding between MDM2 and the *p53* mRNA does not inhibit auto-ubiquitination or ubiquitination of MDMX under genotoxic stress conditions (140). This is consistent with previous reports showing a decrease in MDMX levels under DNA damage conditions (146). Hence, the binding of the *p53* mRNA to MDM2 controls the substrate specificity of MDM2 and activates p53 by inducing its synthesis while preventing its degradation.

### Controlling the stability of the encoded protein

Synonymous mutations can affect the function of the encoded protein. This has been described in prokaryotes as well as in eukaryotes and has been linked to changes in ‘fast’ and ‘slow’ codons that affect the rate of translation and the folding of the nascent protein (13,80,147). The *p53* mRNA provides an example of an alternative mechanism whereby an mRNA can affect the encoded protein. An increase in *p53* mRNA - MDM2 affinity by introducing synonymous mutations in *p53* at codons 17, 18 and 19 stimulates p53’s rate of synthesis and its rate of degradation in an MDM2-dependent fashion under normal conditions (108). This suggests that MDM2-dependent synthesis of p53 allows MDM2 access to the nascent p53 protein, which was supported by p53 polysome pull-down assays. A role for the *p53* mRNA in controlling the stability of the encoded protein is also implicated by the observation that the accumulation of wild type p53 protein following DNA damage does not take place when expressed from a non-MDM2 binding *p53* mRNA that carries a synonymous cancer-derived mutation in codon 22 (L22L) (106,108,148) or to other two single silent cancer-derived p53 mutations V10V and P36P (149,150) (Table 2). Hence, the increase in MDM2 - *p53* mRNA affinity during DNA damage does not result in an increase in p53 degradation. The explanation for why an increase in MDM2 – *p53* mRNA affinity during DNA damage, but not under normal conditions, results in an increase in p53 levels can be explained by the observation that during DNA damage, MDM2 brings the ATM kinase to the p53 polysome where it phosphorylates the nascent p53 protein (151). This event prevents MDM2 from binding to p53 and, thus allowing MDM2 to stimulate p53 synthesis without targeting p53 for degradation (Figure 3). The trafficking of ATM to the p53 polysome also requires MDM2’s interaction with ribosomal factors and mutations in MDM2 that inhibit the binding to RPL5 and RPL11 prevents ATM-mediated phosphorylation of the nascent p53 (151). According to the intrinsic disordered protein model, post-translational modifications and consequent protein–protein interactions are important to differentiate protein activity. This model indicates that the first event in this cascade can depend on the encoding mRNA and happen while the protein is being synthesized.

## CONCLUSION AND PERSPECTIVES

The role of synonymous mutations in controlling protein function is usually attributed to a switch between ‘fast’ and ‘slow’ codons that can affect translation elongation rate and how the protein is folded. The example presented here of how a synonymous mutation affects the encoded protein by causing changes in the RNA structure and protein binding illustrates an alternative role for mRNAs in controlling the encoded protein. Hence, it can be difficult to deduce that changes in the encoded protein by synonymous mutations are due to changes in ‘fast vs slow’ codons, or by affecting an RNA-protein interaction (152–157). Mutations affecting the peptide sequence do not rule out a role for the mRNA in causing a biological effect. For example, the importance of phosphorylation events on residues in the BOX-1 region of p53 has been somewhat controversial. One study suggested the importance of serine 20 based on alanine substitution (158). But the same effect on p53 activity was observed by maintaining the wild type amino acidic sequence while altering nucleotides to mimic the changes of the alanine mutation in the RNA structure (108). Considering how frequently mutations are used to determine the role of single amino acids or protein domains, there are likely to be other examples where cell biological effect attributed to the peptide sequence in fact are due to changes in the encoding mRNA.

The ATM kinase plays a key role in the DNA damage response and in activating p53. It is interesting that ATM acts both on the folding of the nascent *p53* mRNA and on the nascent p53 protein and how these two events are interlinked. It will be interesting to know if this is unique for ATM - p53, or if it represents a broader general mechanism for synchronizing synthesis and degradation by a signalling pathway. The concept of releasing a protein from the ribosome that is predestined for a certain activity is intuitively attractive. p53 with its over 300 cellular interacting factors that are regulated by over 60 different post-translational modifications is a good model system for such studies.

The *p53* mRNA acts as a ligand to control the function of MDM2 and the substrate specificity of its E3 ligase activity. This is another molecular mechanism that has not yet been described for other mRNAs, but one would expect that also this concept is not unique.

The DNA damage induced accumulation of p53 is well documented in many cell types and the role of increased translation is evident from the association of p53 with polysomes. Many studies have identified some mechanism participating in *p53* mRNA regulation with impact on localization, stability, translational rate etc. but how these different interactions are orchestrated, and to which extend they cooperate, needs further studies. For instance, how do *p53* mRNA binding factors such as MDM2, MDMX and ATM interplay with general RNA binding proteins such as HuR to achieve stress-induced p53 activation?

The mammalian p53 family consists of p53, p63 and p73 that have evolved from a single ancestral p53/p63/p73. While p53 plays a key role in cellular homeostasis and stress responses, p63 and p73 have important roles in development (159–163). As of today, little is known if/how the mRNAs of p63 and p73 also play a role in regulating the activity of respective encoded proteins. The observation that RBM38 interacts with the p63 *and* p53 messages suggest that co-regulated translation of these messages might take place. Co-regulated translation of mRNAs in stress response pathways is yet a relatively unexplored phenomenon.
